# Effect of Ce Treatment on the Austenite Grain Growth Behavior of High-Strength Low-Alloy Steel During Heating Process Before Rolling

**DOI:** 10.3390/ma19071343

**Published:** 2026-03-28

**Authors:** Fei Huang, Jing Li, Bin Lu

**Affiliations:** 1 State Key Laboratory of Advanced Metallurgy, University of Science and Technology Beijing, 30 Xueyuan Road, Haidian District, Beijing 100083, China; huangfei19960923@163.com; 2 Technical Center of Inner Mongolia Baotou Steel Union Co., Ltd., Baotou 014010, China; lubinbt@126.com; 3 Beijing Baotou Steel Technology Co., Ltd., Beijing 100083, China

**Keywords:** Ce treatment, high-strength low-alloy steel, grain growth, in situ observation, precipitated phase, pinning force

## Abstract

By adding Ce to high-strength low-alloy steel, the effects of heating parameters and Ce on grain growth were examined through in situ observation and dynamic analysis of grain growth behavior during heating, combined with precipitated phase analysis and pinning force calculations. In situ observation of the heating process revealed the behavior of grain growth and grain boundary migration in real time, providing an intuitive and accurate illustration of the effect of Ce on grain growth behavior. The mechanism of Ce’s role in refining austenite grains was clarified. The results revealed that at 1050 °C, Ce had little effect on grain growth. Ce delayed the grain coarsening temperature from 1050–1150 °C to 1150–1250 °C, resulting in grain refinement. The predicted results from the established dynamic model were consistent with the grain growth process, demonstrating high predictive accuracy. After Ce treatment, the activation energy for grain growth increased from 172.058 to 193.703 kJ/mol, representing a 12.58% rise, rendering grain growth more difficult. Within the holding temperature range, small spherical Nb-rich (Nb, Ti)(C, N) and large rectangular Ti-rich (Nb, Ti)(C, N) existed. The addition of 0.0070% Ce delayed the dissolution of Nb-rich carbonitrides. Finer precipitated phases and high-melting-point, fine Ce_2_O_2_S and CeAlO_3_ inclusions at grain boundaries provided greater pinning force, inhibiting grain growth.

## 1. Introduction

High-strength low-alloy (HSLA) steel offers high strength, high toughness, and excellent weldability [1,2,3], effectively reducing steel weight and utilization while significantly cutting resource and production energy consumption. With its low alloy content and mature manufacturing processes, it achieves a balance between cost and performance, making it widely used in bridges, ships, pressure vessels, and other applications [4,5,6].

HSLA steel requires heating and holding prior to thermo-mechanical control process (TMCP) rolling to achieve full austenitizing and ensure uniform microstructure. During the heating and holding process, the grain size of the austenite primarily depends on the holding temperature and holding time [7,8]. Neither should be excessively high, as this would cause excessive grain growth [9], making subsequent grain refinement difficult after rolling. Nor should they be excessively low, as this would result in poor grain size uniformity, adversely affecting subsequent rolling operations. With the development trend toward large-scale and lightweight equipment manufacturing, the performance requirements for HSLA steels are increasingly demanding [4,10]. Achieving fine austenite grains in the microstructure is an effective approach to enhancing the comprehensive properties of materials [11,12,13]. Due to the heredity of the microstructure, obtaining fine austenite grains during the heating and holding process facilitates the refinement of grains in the finished products. Therefore, studying grain growth behavior during the heating process before rolling is of significant importance.

Rare earth can play a role in purifying molten steel, modifying inclusions and microalloying [14,15,16], and also affect the growth of austenite grains. Liu et al. [17] added rare earth elements to X80 pipeline steel and found that the rare earths suppressed abnormal grain growth in the austenite, resulting in fine grain sizes. Yang et al. [18] demonstrated that adding rare earth oxides to flux cored wires for hardfacing the workpieces of medium–high carbon steel enabled LaAlO_3_ to act as a heterogeneous nucleation site for γ-Fe, thereby refining the primary austenite grains in the hardfacing layer and reducing their average grain size. Song et al. [19] indicated that when the holding temperature of high niobium-containing rare earth steel was below 1220 °C, both the austenite grain size and growth rate were small, demonstrating excellent resistance to coarsening at high temperature. Zhou et al. [20] applied La treatment to H13 steel, increasing the quantity of undissolved carbides V_8_C_7_, refining the carbides, and generating high-melting-point La_2_O_2_S. This enhanced the pinning effect at grain boundaries, effectively inhibiting austenite grain growth and resulting in a more uniform grain size distribution. Jiang et al. [21] treated ultra-high-strength steel with Ce and found that Ce could refine the austenite grain size. This was primarily due to the pinning effect of fine Ce-containing inclusions on the grain boundaries, which inhibited grain growth.

Due to the refining effect of rare earth elements on austenite grain size, Ce treatment was applied to the experimental HSLA steel. Our previous research revealed that adding 0.0070% Ce to HSLA steel resulted in a fine, uniform solidification microstructure and reduced element segregation [22]. This had a beneficial effect on inclusions and precipitated phases, and is thereby favorable for austenite grain growth behavior during the heating process. Thus, the Ce content in the experimental HSLA steel was determined as 0.0070%. Currently, grain growth behavior during the heating and holding process is typically analyzed indirectly through room-temperature microstructures [23,24], but real-time observation of grain growth and grain boundary migration processes remains unavailable. A high temperature confocal laser scanning microscope (HTCLSM) provides real-time continuous observation of high-temperature phase transformation processes [25], making it a powerful tool for in situ observation of grain growth behavior [26,27]. Therefore, this study employed HTCLSM to conduct dynamic in situ observation of grain growth behavior during the heating process of Ce-treated HSLA steel. The influence of heating parameters and Ce on grain growth was analyzed. The grain growth dynamic models for HSLA steel were established, clarifying the detailed mechanisms of precipitated phases and Ce in grain growth.

## 2. Experimental

### 2.1. Materials Preparation

Experimental high-strength low-alloy steel was smelted in the 50 kg vacuum induction furnace using industrial pure iron and alloy materials. Ce treatment was performed using the Ce-Fe alloy containing 30% Ce, and the steel was cast into ingots with dimensions of 150 mm × 150 mm × 300 mm. The chemical compositions of experimental steels are listed in Table 1. After 0.0070% Ce treatment, the O and S contents in steel were reduced by 0.0010% and 0.0017%, respectively.

### 2.2. In Situ Observation and Analysis of Grain Growth Behavior

**Figure 1:**
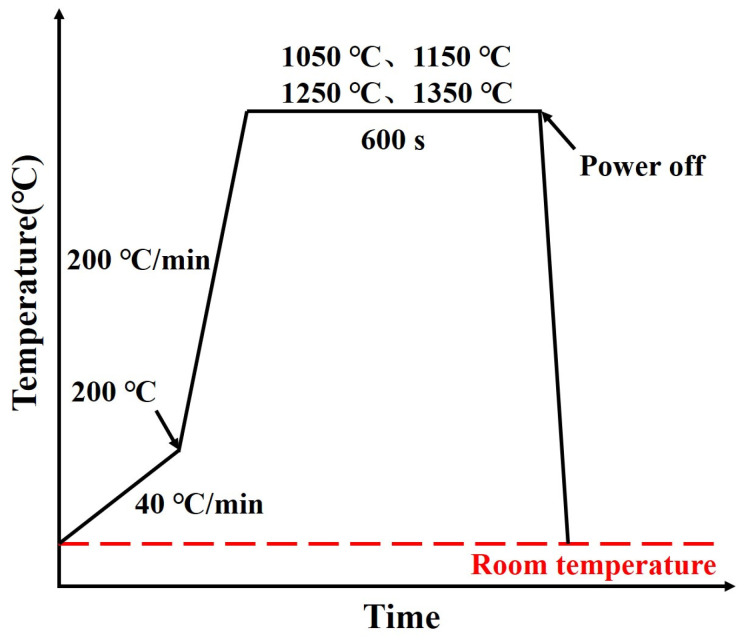
Experimental procedure diagram for in situ observation of grain growth behavior during heating process.

The study involved cutting 10 mm thick specimens from the height direction of experimental steel ingots, then extracting specimens with dimensions of 7 mm in diameter and 3 mm in height at the 1/4 diagonal of their cross sections. After grinding and polishing, these specimens were prepared for in situ observation experiments. HTCLSM enabled dynamic observation of grain growth behavior during the heating process, and the specific experimental procedure is illustrated in Figure 1. Firstly, the specimens were heated to 200 °C at 40 °C/min after high purity argon gas was introduced and vacuumized. Subsequently, they were heated at 200 °C/min to 1050, 1150, 1250, and 1350 °C, respectively, with each temperature held for 600 s. Upon completion of the experiment, power was immediately cut off and the specimens cooled to room temperature. During the experiment, the growth behavior of grains was observed and recorded in real time through the imaging system. Using Image-Pro Plus 6.0 software to process real-time images captured during in situ observation of the heating and holding process, the grain sizes at each holding temperature and holding time were determined.

### 2.3. Observation and Characterization of Precipitated Phases During Heating Process

Samples with dimensions of 10 mm × 10 mm × 10 mm were also cut from the 1/4 diagonal of cross sections in 10 mm thick specimens, and the heating and holding experiments were carried out using the KSL-1700X high temperature resistance furnace (MTI Corporation, Richmond, CA, USA). The samples were heated to 1050, 1150, 1250 and 1350 °C at the heating rate of 10 °C/min for 0, 120, 360 and 600 s, respectively, and then water quenched to room temperature. After the experiment, the polished specimens were etched with 4 vol% nitric acid alcohol solution for approximately 1 min. After carbon spraying, a square of 1.5 mm × 1.5 mm was carved with a knife and soaked in FeCl_3_ hydrochloric acid alcohol solution to remove the carbon film. The carbon films were fished out using copper mesh and dried in an oven to produce carbon replica specimens. Subsequently, the morphology and distribution of precipitated phases in the specimens were observed via transmission electron microscope (TEM, JEM-2100F, JEOL, Tokyo, Japan), and their types were analyzed using an energy dispersive spectrometer (EDS) and diffraction patterns. Additionally, multiple fields of view were randomly selected, and the sizes of precipitated phases were measured utilizing Image-Pro Plus 6.0 software.

## 3. Results and Discussion

### 3.1. In Situ Observation of Austenite Grain Growth Behavior

#### 3.1.1. Effect of Holding Temperature on Austenite Grain Growth

**Figure 2:**
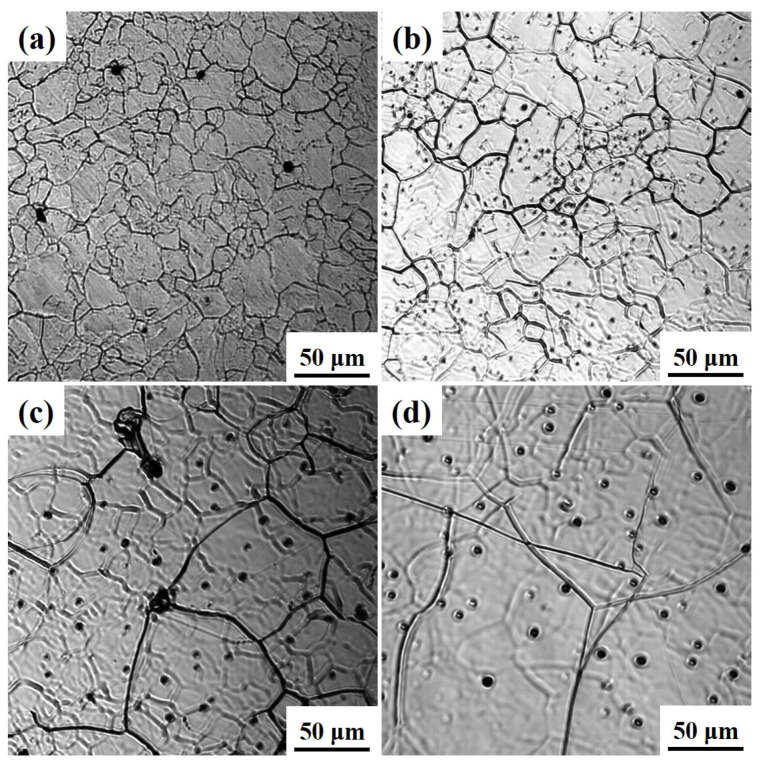
Austenite grain morphology of Ce-free steel after holding at different holding temperatures for 360 s: (**a**) 1050 °C; (**b**) 1150 °C; (**c**) 1250 °C; (**d**) 1350 °C.

During the heating process, the influence of heating conditions on grain growth in the experimental steel was consistent. Taking Ce-free steel as an example, Figure 2 shows the austenite grain morphology of Ce-free steel at 1050, 1150, 1250, and 1350 °C after holding for 360 s. As the temperature increased, the grains of Ce-free steel gradually transformed from an irregular shape to nearly hexagonal. At 1050 °C, the grain size distribution was uneven, with an overall fine grain size. At 1150, 1250, and 1350 °C, the grain size progressively increased with rising temperature, becoming significantly larger than that at 1050 °C.

**Figure 3:**
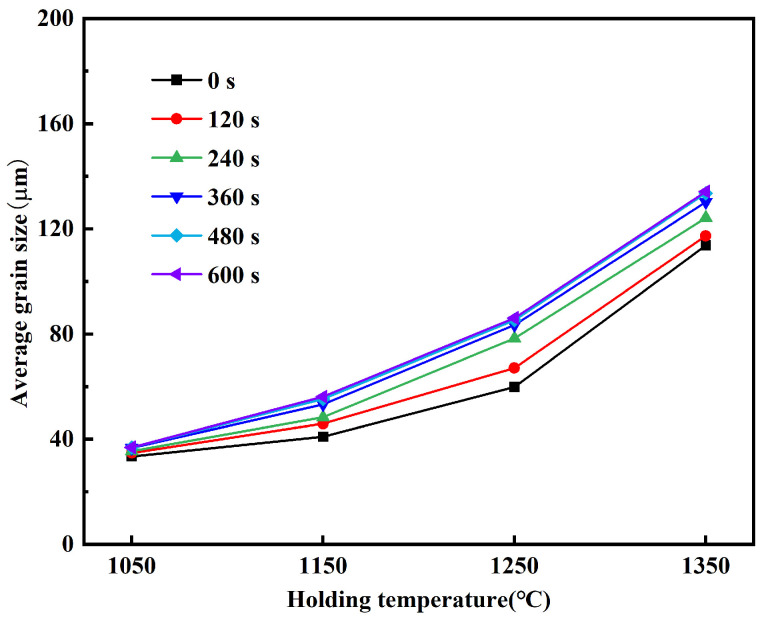
The variation in average austenite grain size with holding temperature in Ce-free steel.

The average austenite grain size of Ce-free steel at different holding temperatures was measured, with the results shown in Figure 3. The average grain sizes under different heating conditions were all obtained by statistically analyzing more than 50 grains. At the same holding time, the higher the holding temperature, the larger the grain size of Ce-free steel, with significant increases in size across all temperatures. At a holding time of 360 s, the average grain sizes at 1050, 1150, 1250, and 1350 °C were 36.69, 53.27, 83.45, and 130.21 μm, respectively, representing increases of 45.19%, 56.65%, and 56.03%. As the holding temperature increased, the grain size of Ce-free steel grew rapidly, indicating its poor resistance to coarsening.

#### 3.1.2. Effect of Holding Time on Austenite Grain Growth

**Figure 4:**
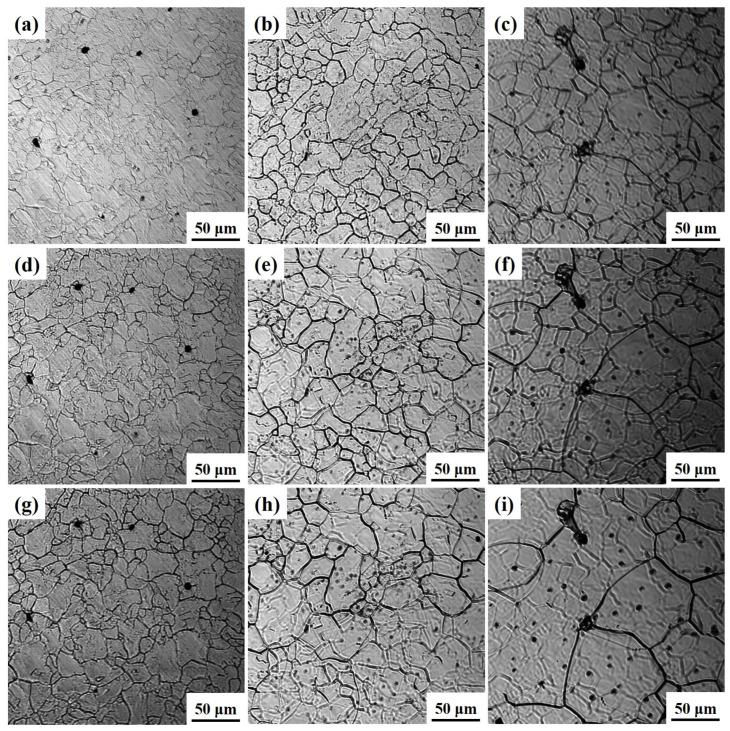
Austenite grain morphology of Ce-free steel after holding at 1050 (**a**,**d**,**g**), 1150 (**b**,**e**,**h**), and 1250 °C (**c**,**f**,**i**) for different times: (**a**–**c**) 0 s; (**d**–**f**) 240 s; (**g**–**i**) 480 s.

The austenite grain morphology of Ce-free steel at 1050, 1150, and 1250 °C after different holding times is shown in Figure 4. At 1050 °C, the grain size was relatively small but exhibited poor uniformity. When held for 480 s, grain growth proceeded at a relatively slow rate, with no significant difference in grain size observed. At 1150 °C, grain size increased compared to 1050 °C, with significantly improved uniformity. As holding time increased, grains merged and grew into larger sizes. When holding time was below 240 s, grain growth was slow, whereas at 480 s, grains grew substantially. At 1250 °C, grains grew rapidly with increasing holding time.

**Figure 5:**
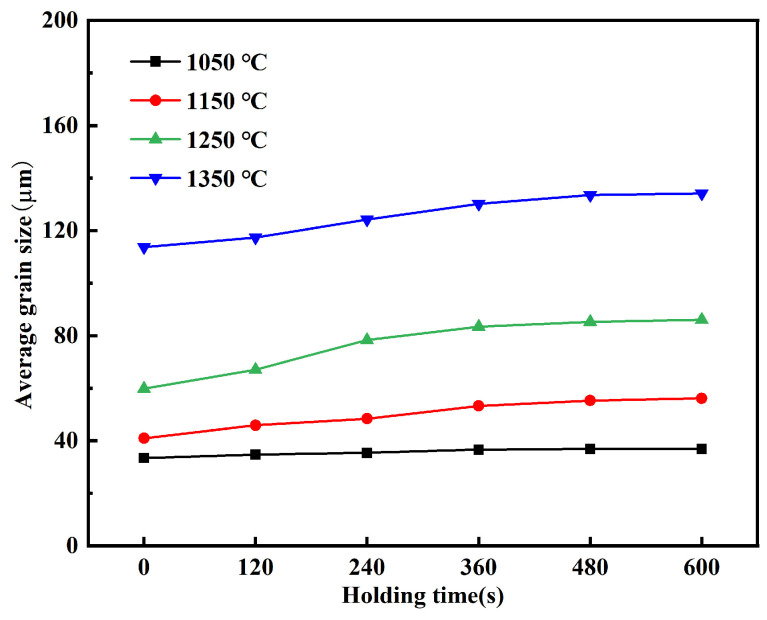
The variation in average austenite grain size with holding time in Ce-free steel.

Figure 5 shows the average austenite grain size of Ce-free steel at 1050, 1150, 1250, and 1350 °C for different holding times. At 1050 °C, as the holding time increased from 0 to 600 s, the grains of Ce-free steel remained relatively fine. The average grain size increased from 33.47 to 36.91 μm, showing a negligible increase. At holding temperatures of 1150, 1250, and 1350 °C, grain size increased rapidly with holding time. During the holding time increase from 0 to 600 s, the average grain size increased by 15.21, 26.29, and 20.42 μm at these three temperatures, respectively. At the holding temperature of 1050 °C, the grain size of Ce-free steel showed no significant change with increasing holding time, exhibiting only a slight increase. At this temperature, the steel demonstrated good resistance to grain coarsening. When the temperature exceeded 1050 °C, grain coarsening accelerated rapidly with extended holding time, though the grain growth rate progressively slowed down.

#### 3.1.3. Effect of Ce on Austenite Grain Growth

**Figure 6:**
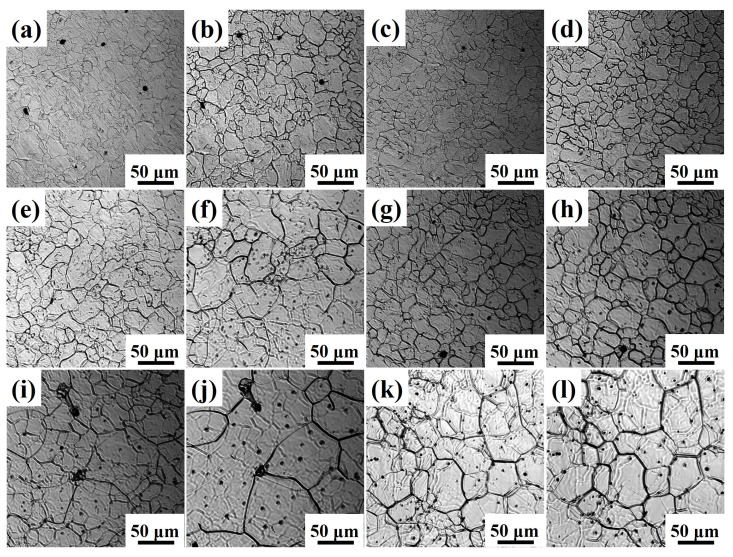
Austenite grain morphology of Ce-free (**a**,**b**,**e**,**f**,**i**,**j**) and Ce-treated steel (**c**,**d**,**g**,**h**,**k**,**l**) under different heating conditions: (**a**,**c**,**e**,**g**,**i**,**k**) 60 s; (**b**,**d**,**f**,**h**,**j**,**l**) 600 s; (**a**–**d**) 1050 °C; (**e**–**h**) 1150 °C; (**i**–**l**) 1250 °C.

To analyze the effect of Ce treatment on austenite grain growth, the austenite grain morphology of Ce-free and Ce-treated steel under different heating conditions was compared, as shown in Figure 6. Without Ce addition, the grain size rapidly increased with rising holding temperature. At temperatures exceeding 1050 °C, grain coarsening became severe as the holding time increased. At 1050 and 1150 °C, the grains in Ce-treated steel remained relatively fine. When heated to 1250 °C or higher, the grains in Ce-treated steel coarsened significantly. At 1050 and 1150 °C, the grain size of Ce-treated steel increased slowly with increasing holding time, showing no significant change in size. At 1250 °C and above, grain growth in Ce-treated steel accelerated rapidly with extended holding time. Under identical heating conditions, the austenite grains in Ce-treated steel were finer than those in Ce-free steel.

**Figure 7:**
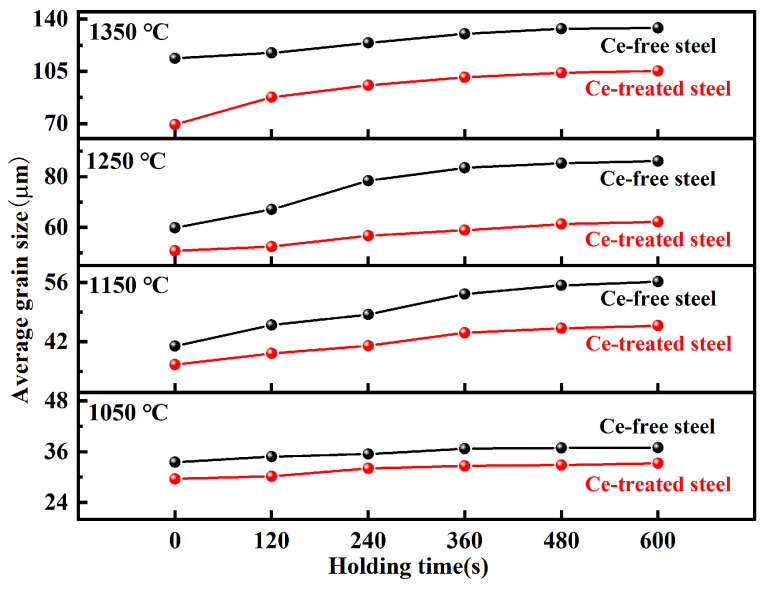
Comparison of average austenite grain sizes in Ce-free and Ce-treated steel under different heating conditions.

Figure 7 presents the comparison of average austenite grain sizes for Ce-free and Ce-treated steel under different heating conditions. At the holding temperature of 1050 °C, the grain size increase in Ce-free and Ce-treated steel was relatively small with extended holding time. After 600 s of holding, the average grain sizes were 36.91 and 33.22 μm, respectively, showing little difference. At this temperature, Ce had no apparent effect on grain growth. At 1150 °C, grain size in Ce-free steel increased significantly with extended holding time, while Ce-treated steel grains grew slowly, widening the size gap. After 600 s of holding, the average grain size of Ce-treated steel decreased by 10.42 μm compared to Ce-free steel. At elevated temperatures of 1250 and 1350 °C, grain coarsening occurred rapidly in both experimental steels, but Ce-treated steel exhibited finer grains. After holding for 600 s, the average grain sizes differed by 23.89 and 28.85 μm, respectively.

The grain coarsening temperature of Ce-free steel ranged from 1050 to 1150 °C, while that of Ce-treated steel fell between 1150 and 1250 °C. Rare earth Ce suppressed austenite grain growth during heating, and delayed the grain coarsening temperature.

### 3.2. Dynamic Analysis of Austenite Grain Growth Process

#### 3.2.1. Establishment of Dynamic Model for Austenite Grain Growth

The Beck model [28], Anelli model [29], and Hillert model [30] are commonly used grain growth models, as shown in Equations (1)–(3), respectively.

(1) $$D_{t}=Kt^{n}$$

where *D_t_* is the average grain size at holding time *t* (µm), *t* is the holding time (s), *K* and *n* are constants.

(2) $$D_{t}=Bt^{m}\exp\left(-\frac{Q}{RT}\right)$$

where *T* is the holding temperature (K), *Q* is the grain growth activation energy (J/mol), *R* is the gas constant (8.314 J/(mol·K)), and *B* and *m* are constants.

(3) $${D_{t}}^{2}-{D_{0}}^{2}=At\exp\left(-\frac{Q}{RT}\right)$$

where *D*_0_ is the initial average grain size at the beginning of heating (µm), and *A* is a constant.

**Figure 8:**
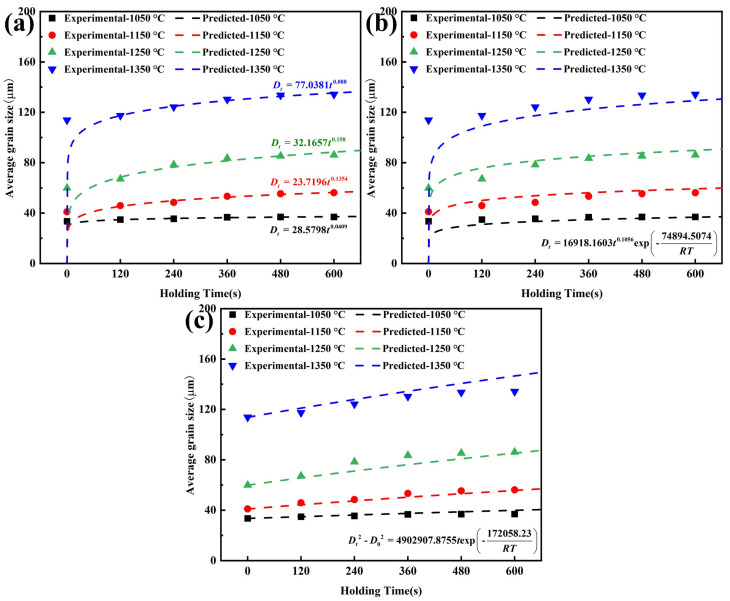
Experimental data and model fitting curves for grain size in Ce-free steel during heating: (**a**) Beck model; (**b**) Anelli model; (**c**) Hillert model.

Taking Ce-free steel as an example to illustrate the model fitting results, as shown in Figure 8, for the Beck and Anelli models, the predicted values of Ce-free steel grain size deviated significantly from experimental values during shorter holding times. As the holding time increased, the discrepancy between the predicted and experimental values noticeably decreased, and the degree of agreement improved. Under different heating conditions, the predicted results from the Hillert model closely matched the experimental values, demonstrating high predictive accuracy.

#### 3.2.2. Model Accuracy Analysis

The decision coefficient (R^2^), root mean square error (RMSE), and symmetric mean absolute percentage error (SMAPE) were introduced to further evaluate the accuracy of grain growth models for Ce-free steel, as shown in Equations (4)–(6).

(4) $$R^{2}=1-\frac{\sum_{i=1}^{n}{\left(D_{\exp,i}-D_{p\mathrm{red},i}\right)}^{2}}{\sum_{i=1}^{n}{\left(D_{\exp,i}-\bar{D_{\exp}}\right)}^{2}}$$

(5) $$\mathrm{RMSE}=\sqrt{\frac{1}{n}\sum_{i=1}^{n}{\left(D_{\mathrm{pred},i}-D_{\exp,i}\right)}^{2}}$$

(6) $$\mathrm{SMAPE}=\frac{100\%}{n}\sum_{i=1}^{n}\frac{\left|D_{\mathrm{pred},i}-D_{\exp,i}\right|}{\left(\left|D_{\mathrm{pred},i}\right|+\left|D_{\exp,i}\right|\right)/2}$$

where *D*_exp_ is the experimental value of grain size (µm), *D*_pred_ is the predicted value of grain size (µm), $\bar{D_{\exp}}$ is the average experimental value of grain size (µm), and *n* is the number of data points.

**Figure 9:**
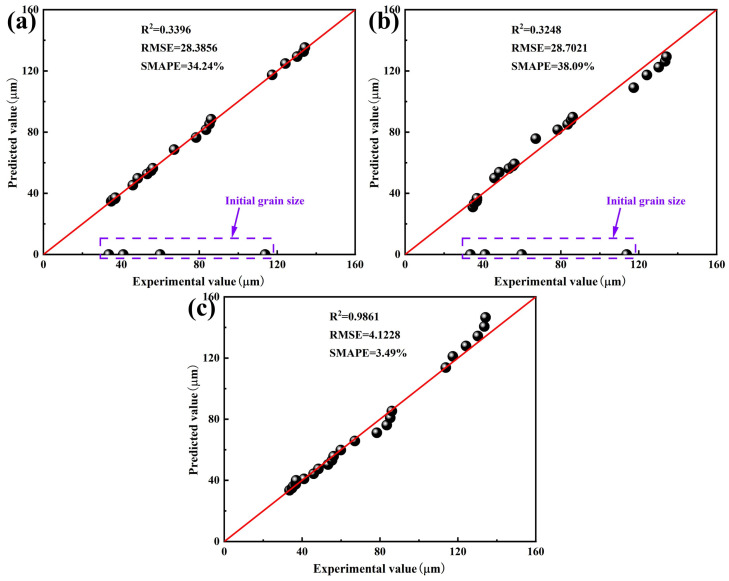
Correlation between predicted values from the Ce-free steel grain growth models and experimental data: (**a**) Beck model; (**b**) Anelli model; (**c**) Hillert model.

The larger the R^2^ value, the smaller the RMSE and SMAPE values, indicating the higher degree of agreement between the model predictions and experimental data, and thus a more accurate model. Evaluation indexes for the grain growth models of Ce-free steel were calculated, and the correlation between model predictions and experimental data was compared, as shown in Figure 9. The Beck and Anelli models showed significant prediction deviations for initial grain sizes at various holding temperatures. Their R^2^ values were 0.3396 and 0.3248, respectively, with RMSE values of 28.3856 and 28.7021, and SMAPE values of 34.24% and 38.09%, indicating low prediction accuracy. In contrast, all data points in the Hillert model were located near the optimal regression fitting line. The R^2^ value improved to 0.9861, while the RMSE and SMAPE values decreased to 4.1228 and 3.49%, respectively, demonstrating high prediction accuracy.

The Beck model focused solely on the effect of holding time on grain growth behavior during heating, neglecting the influence of holding temperature and initial austenite grain size. The prediction of grain size during the initial holding stage exhibited significant inaccuracies. The Anelli model comprehensively considered the effects of holding temperature and holding time on grain growth, yet it still neglected the initial grain size, resulting in no improvement in prediction accuracy. The Hillert model assumed that grain growth was driven by interfacial energy, focusing on interface-controlled kinetics. It took into account the combined effects of holding temperature, holding time and initial grain size, thereby accurately describing the evolution of average grain size in homogeneous materials under steady-state conditions. Furthermore, the feasibility of mixed-control and abnormal-growth descriptions was analyzed. When the effects of solute elements and precipitated phases on grain boundary migration were comparable, the mixed-control model proved to be relatively reliable. At lower temperatures, grain boundary migration was primarily influenced by the dragging effect of solute elements. At higher temperatures, the driving force for grain boundary growth was determined by the pinning force of precipitated phases, with solute elements having negligible influence [31,32]. The heating process operated at relatively high temperatures ranging from 1050 to 1350 °C, mainly considering the effect of precipitated phases on grain growth. Consequently, the adoption of mixed-control model was unnecessary. Given that the phenomenon of abnormal grain growth in Ce-treated steel during the heating process was not evident, the abnormal-growth model was unable to provide a unified analysis of the grain growth behavior in the two experimental steels. Ultimately, the dynamic model for austenite grain growth during the heating process of Ce-free steel was established, as shown in Equation (7).

(7) $${D_{t}}^{2}-{D_{0}}^{2}=4902907.8755t\exp\left(-\frac{172058.23}{RT}\right)$$

#### 3.2.3. Dynamic Analysis of the Effect of Ce on Grain Growth Behavior

**Figure 10:**
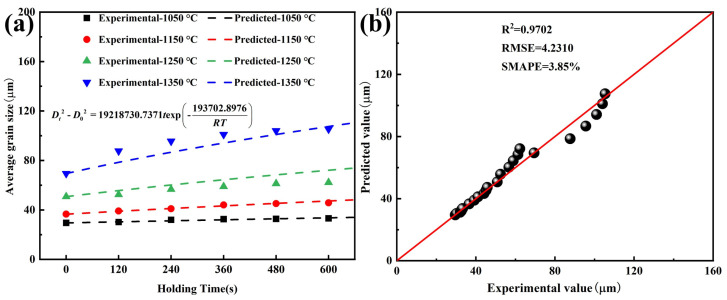
Fitting results and correlation of grain growth dynamic model for Ce-treated steel: (**a**) Hillert model fitting results; (**b**) correlation between predicted and experimental values.

Based on the above analysis, the Hillert model was employed to conduct dynamic analysis of grain growth process in Ce-treated steel. The model fitting results and correlation are shown in Figure 10. Throughout the entire heating and holding process, R^2^, RMSE, and SMAPE values for the Hillert model were 0.9702, 4.2310, and 3.85%, respectively, indicating high correlation. The predicted values closely matched the experimental values. Therefore, the grain growth dynamic model for Ce-treated steel was determined, given by Equation (8).

(8) $${D_{t}}^{2}-{D_{0}}^{2}=19218730.7371t\exp\left(-\frac{193702.8976}{RT}\right)$$

Based on the dynamic model fitting results from Equations (7) and (8), the grain growth activation energies for Ce-free and Ce-treated steel were 172.058 and 193.703 kJ/mol, respectively. The addition of 0.0070% Ce increased the activation energy for grain growth in steel, making grain growth more difficult at high temperature and raising the grain coarsening temperature. This effectively suppressed the tendency for austenite grain growth during the heating process. Thus, at the same holding temperature and holding time, the average grain size of Ce-treated steel was smaller than that of Ce-free steel.

### 3.3. Analysis of Austenite Grain Growth Behavior

#### 3.3.1. Analysis of the Type, Morphology, and Size of Precipitated Phases During the Heating Process

**Figure 11:**
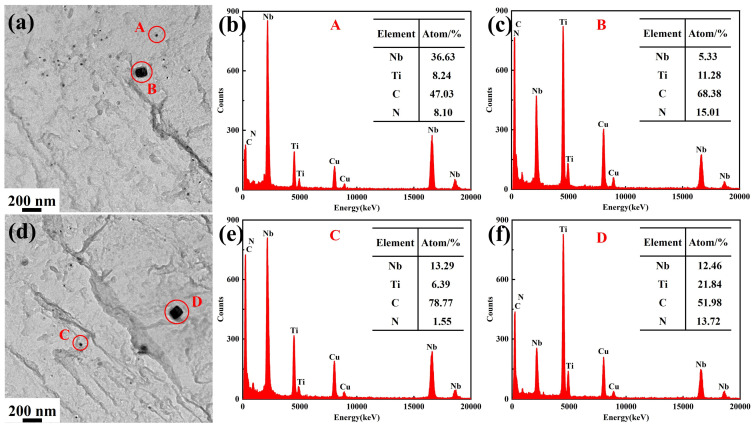
TEM images and corresponding EDS spectra of precipitated phases in the specimens after holding at 1050 °C for 360 s: (**a**,**d**) morphology of precipitated phases; (**b**,**c**,**e**,**f**) EDS spectra of precipitated phases.

Figure 11 shows the TEM images and EDS results of precipitated phases after heating. Due to the use of copper mesh for preparing carbon replica specimens, a certain amount of Cu element existed in the EDS spectra. The precipitated phases all consisted of Nb, Ti, C, and N elements, but exhibited distinct differences in morphology and size, primarily categorized into two types. One type comprised fine spherical or ellipsoidal precipitated phases (A and C), which, according to EDS analysis, contained higher Nb content, as shown in Figure 11b,e. The other type consisted of larger rectangular precipitated phases (B and D), which contained higher Ti content, as shown in Figure 11c,f.

**Figure 12:**
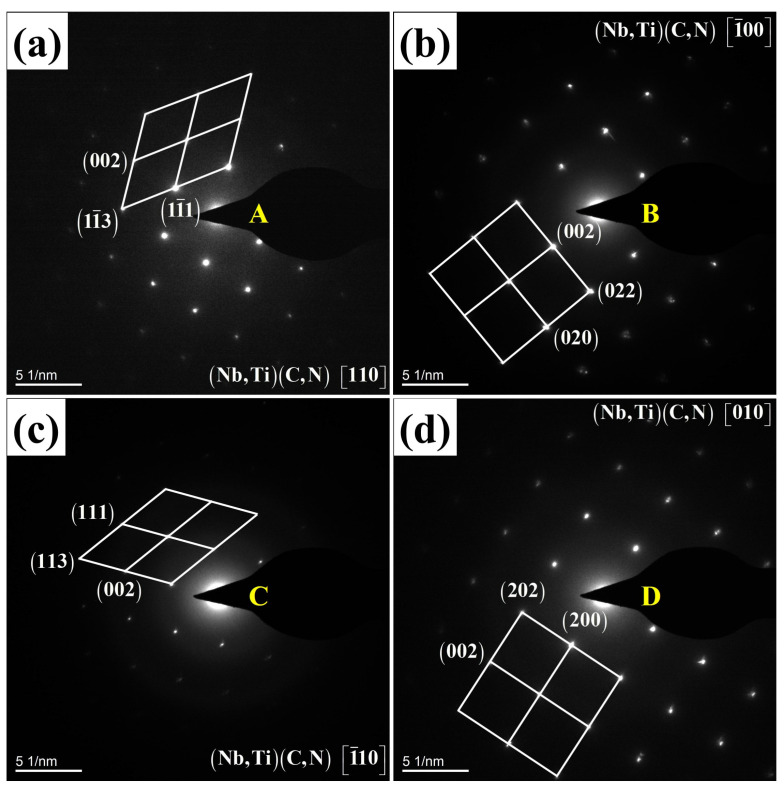
The diffraction patterns and calibration results of precipitated phases in the specimens after holding at 1050 °C for 360 s: (**a**,**c**) Nb-rich (Nb, Ti)(C, N); (**b**,**d**) Ti-rich (Nb, Ti)(C, N).

To identify the types of precipitated phases, further analysis was conducted using selected area electron diffraction, as shown in Figure 12, where A–D correspond to the precipitated phases shown in Figure 11. Both precipitated phases exhibited face-centered cubic structures of (Nb, Ti)(C, N). Consequently, the smaller spherical precipitated phase was Nb-rich (Nb, Ti)(C, N), while the larger rectangular precipitated phase was Ti-rich (Nb, Ti)(C, N), consistent with the findings of Akhlaghi et al. [33].

The heating process is typically accompanied by coarsening and dissolution behavior of precipitated phases. When the dissolution temperature of precipitated phase is lower than the holding temperature, it gradually dissolves. Conversely, if the holding temperature does not reach the dissolution temperature of precipitated phase, Ostwald ripening occurs, where smaller-sized precipitates dissolve while larger-sized ones coarsen [34].

**Figure 13:**
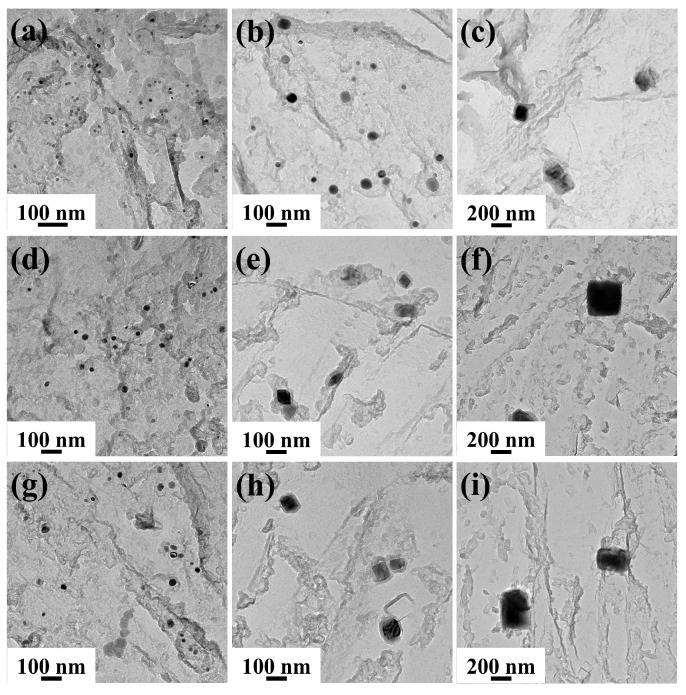
Morphology of precipitated phases in Ce-free steel after holding at 1050 (**a**,**d**,**g**), 1150 (**b**,**e**,**h**), and 1250 °C (**c**,**f**,**i**) for different times: (**a**–**c**) 0 s; (**d**–**f**) 360 s; (**g**–**i**) 600 s.

**Figure 14:**
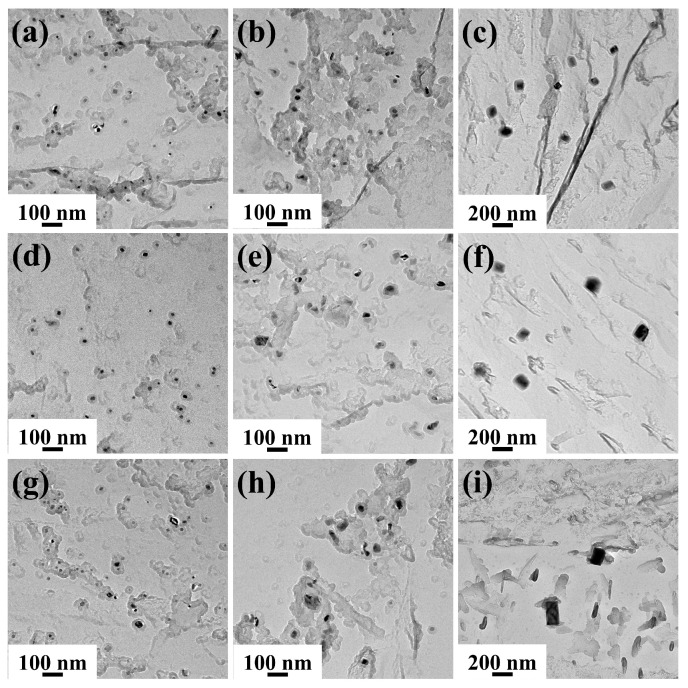
Morphology of precipitated phases in Ce-treated steel after holding at 1050 (**a**,**d**,**g**), 1150 (**b**,**e**,**h**), and 1250 °C (**c**,**f**,**i**) for different times: (**a**–**c**) 0 s; (**d**–**f**) 360 s; (**g**–**i**) 600 s.

Figure 13 and Figure 14 show the TEM images of precipitated phases in Ce-free and Ce-treated steel under different heating conditions, respectively. At 1050 °C, two types of precipitated phases existed in Ce-free steel, featuring small dimensions and uniform distribution. At 1150 °C, a small amount of the precipitates began to dissolve and coarsen, decreasing in number and increasing in size. After holding at 360 and 600 s, only larger-sized rectangular Ti-rich (Nb, Ti)(C, N) were observed, with no small spherical Nb-rich carbonitrides found. When heated to 1250 °C or higher, only coarse Ti-rich carbonitrides remained in the matrix. Their quantity decreased sharply while their size increased significantly. A large number of precipitated phases were noticeably dissolved and coarsened. At the same holding temperature, as the holding time increased, the number of precipitated phases decreased and their size increased, whilst they also underwent dissolution and coarsening. The behavior of precipitates in Ce-treated steel during heating was largely consistent with that of Ce-free steel, though some differences were observed. At holding temperatures of 1050 and 1150 °C, both Nb-rich and Ti-rich carbonitrides were present in the matrix as holding time increased. Nb-rich carbonitrides only dissolved completely when the temperature reached 1250 °C. Under identical heating conditions, the precipitated phases in Ce-treated steel were finer in size and more uniformly dispersed compared to those in Ce-free steel.

**Figure 15:**
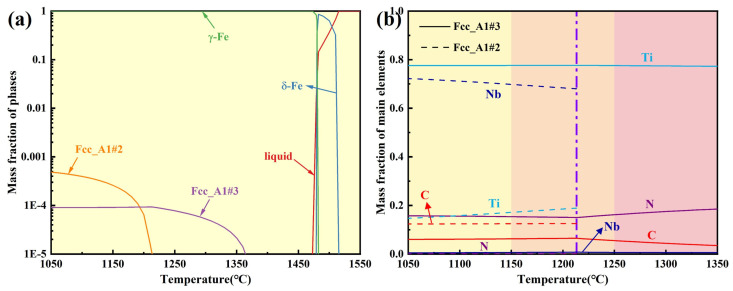
Equilibrium phase diagram for the experimental steel (**a**) and mass fraction of the main elements in precipitated phases over the temperature range of 1050–1350 °C (**b**).

The equilibrium phase diagram for the experimental steel and the mass fractions of main elements in precipitated phases over the temperature range of 1050–1350 °C were calculated using Thermo-Calc 2018a software, as shown in Figure 15. Within the holding temperature range of 1050–1350 °C, the precipitated phases were Fcc_A1#2 and Fcc_A1#3, with Nb, Ti, C, and N as the primary forming elements. Combined with the TEM analysis results in Figure 11 and Figure 12, Fcc_A1#3 was identified as Ti-rich (Nb, Ti)(C, N), while Fcc_A1#2 was Nb-rich (Nb, Ti)(C, N). Without considering the holding process, the complete dissolution temperature of Nb-rich carbonitride was 1213.3 °C, while that of Ti-rich carbonitride exceeded 1350 °C. Since 1250 and 1350 °C exceeded the complete dissolution temperature of Nb-rich carbonitrides, only large-sized, undissolved Ti-rich carbonitrides remained in the matrix after different holding times, consistent with TEM observations. 1050 and 1150 °C were below the complete dissolution temperatures of both precipitated phases. At the initial stage of holding, both phases were present and gradually dissolved and coarsened as the holding time increased. For Ce-free steel held at 1150 °C for 360 s or longer, the Nb-rich carbonitride completely dissolved, whereas the two precipitated phases remained continuously present in the matrix of Ce-treated steel.

The size of precipitated phases during the heating process of Ce-free and Ce-treated steel was determined by calculating the equivalent area diameter, with the calculation method detailed in Equation (9) [35].

(9) $$D=\sqrt{\frac{4A}{\pi }}$$

where *D* is the equivalent area diameter of precipitated phase (nm), and *A* is the measured area of precipitated phase (nm^2^).

**Figure 16:**
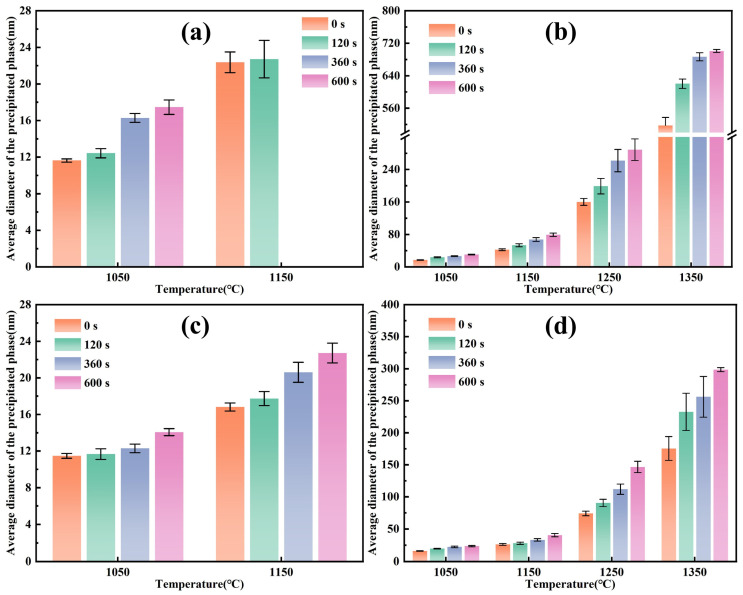
Average diameter of precipitated phases during heating of Ce-free (**a**,**b**) and Ce-treated (**c**,**d**) steel: (**a**,**c**) Nb-rich (Nb, Ti)(C, N); (**b**,**d**) Ti-rich (Nb, Ti)(C, N).

The average diameter of precipitated phases was calculated from the statistical results, as shown in Figure 16. Over 30 precipitated phases were selected at random under each heating condition and their diameters were measured. The mean of these measurements was then calculated to obtain the average diameter of precipitated phases. When heated and held below 1250 °C, the average diameter of precipitated phase increased with rising temperature and extended holding time, though the increase was slight. At temperatures of 1250 °C and above, the precipitated phase exhibited significant coarsening, with a substantial increase in size. At holding temperatures of 1050 and 1150 °C, Nb-rich carbonitrides in Ce-free steel completely dissolved when held at 1150 °C for 360 s or longer, whereas the two precipitated phases remained continuously present in Ce-treated steel. Both Nb-rich and Ti-rich carbonitrides in Ce-treated steel were slightly smaller in size than those in Ce-free steel. At holding temperatures of 1250 and 1350 °C, Ti-rich carbonitrides in Ce-treated steel were markedly finer than those in Ce-free steel.

Under identical heating conditions, the average diameter of precipitated phases in Ce-treated steel was smaller than that in Ce-free steel. Adding Ce to steel uniformly refined the as-cast microstructure and narrowed the solidification temperature range [36]. This significantly reduced the segregation of elements forming precipitated phases such as Nb, Ti, and C, decreased the compositional variations across different regions of the matrix, and resulted in uniform solute distribution. During solidification and cooling, reduced compositional variation effectively prevented severe coarsening of precipitated phases due to local element enrichment and insufficient precipitation caused by element depletion, resulting in finer and more uniform size of precipitated phases. Simultaneously, the uniform composition lowered the nucleation energy barrier for precipitated phases, providing more nucleation sites and facilitating their dispersed distribution. Therefore, after adding Ce, the Nb-rich and Ti-rich carbonitrides became finer and more uniformly dispersed. Even after coarsening and dissolution during the heating process, their size remained smaller than that in Ce-free steel.

#### 3.3.2. Mechanism Analysis of Austenite Grain Refinement by Ce During the Heating Process

The precipitated phases retard grain growth during heating by pinning the austenite grain boundaries. Their pinning force on the grain boundaries can be expressed as [37]:

(10) $$P_{Z}=\beta \frac{\gamma f}{r}$$

where *P_Z_* is the pinning force of precipitated phase (Pa), *β* is the dimensionless constant with value set to 12, *f* is the volume fraction of precipitated phase, *r* is the average radius of precipitated phase (m), and *γ* is the austenite interface energy (J/m^2^).

When the carbon content in steel is ≤0.8%, the austenite interface energy can be calculated using Equation (11) [38].

(11) $$\gamma =\left(0.8-0.35\cdot w_{\left[C\right]}^{0.68}\right)$$

where *w*_[C]_ is the carbon content in steel (wt%).

Due to the presence of two precipitated phases during the heating process, the pinning force equation requires optimization:

(12) $$P_{Z}=\beta \gamma \sum_{i=1}^{n}\frac{f_{i}}{r_{i}}$$

where *f_i_* is the volume fraction of precipitated phase *i*, and *r_i_* is the average radius of precipitated phase *i* (m).

To calculate the pinning force, quantitative analysis of the volume fraction *f* of the precipitated phase is required. The precipitated phases observed in carbon replica specimens are largely dependent on the specimen preparation level, and cannot directly reflect the number and distribution of these phases in the matrix, making it impossible to accurately determine the volume fraction of precipitated phases. The volume fraction of precipitated phase during heating process is calculated using the dynamic method proposed by Moon et al. [39], as shown in Equation (13). This method proved more precise than the volume fractions derived from statistical analysis of observations using carbon replica specimens, but it involved several uncertainties. The calculated results depended on the assumptions of the dynamic model and were also influenced by the accuracy of the thermodynamic database. Hence, the calculated volume fractions should be regarded as providing a semi-quantitative trend.

(13) $$f=f_{0}{\left(1-\frac{2\alpha D_{m}t}{r_{0}^{2}}\right)}^{\frac{3}{2}}$$

where *f*_0_ is the initial volume fraction of precipitated phase, α is the dimensionless constant, *D_m_* is the diffusion coefficient of solute atom (m^2^/s), *t* is the holding time (s), and *r*_0_ is the initial size of precipitated phase (m).

The initial volume fraction *f*_0_ of precipitated phase can be obtained by substituting the initial mass fraction calculated from Thermo-Calc into Equation (14) [37]:

(14) $$f_{0}=\frac{1}{2}m_{0}\times \frac{M_{p}/\rho_{p}}{M_{\mathrm{Fe}}/\rho_{\mathrm{Fe}}}$$

where *m*_0_ is the initial mass fraction of precipitated phase, *M*_p_ is the atomic weight of precipitated phase ((Nb, Ti)(C, N) is 166.828), *ρ*_p_ is the density of precipitated phase ((Nb, Ti)(C, N) is 6.67 g/cm^3^), *M*_Fe_ is the atomic weight of Fe with a value of 55.845, and *ρ*_Fe_ is the density of Fe with a value of 7.86 g/cm^3^.

For carbides and nitrides, C and N are interstitial solid solution elements in the iron matrix, while microalloying elements are substitutional solid solution elements. The diffusion activation energy for interstitial solid solution elements is lower than that for substitutional solid solution elements, and their diffusion rates are significantly higher. Therefore, microalloying elements are the controlling elements. The controlling elements in heating process for Nb-rich and Ti-rich carbonitrides are Nb and Ti, respectively. At different holding temperatures, the diffusion coefficients of Nb and Ti atoms in austenite [34] can be calculated using Equations (15) and (16), respectively.

(15) $$D_{Nb-\gamma }=0.83\exp\left(-\frac{266500}{RT}\right)$$

where *D_Nb-γ_* is the diffusion coefficient of Nb atoms in austenite (m^2^/s), *R* is the gas constant (8.314 J/(mol·K)), and *T* is the holding temperature (K).

(16) $$D_{Ti-\gamma }=0.15\exp\left(-\frac{251000}{RT}\right)$$

where *D_Ti-γ_* is the diffusion coefficient of Ti atoms in austenite (m^2^/s).

**Figure 17:**
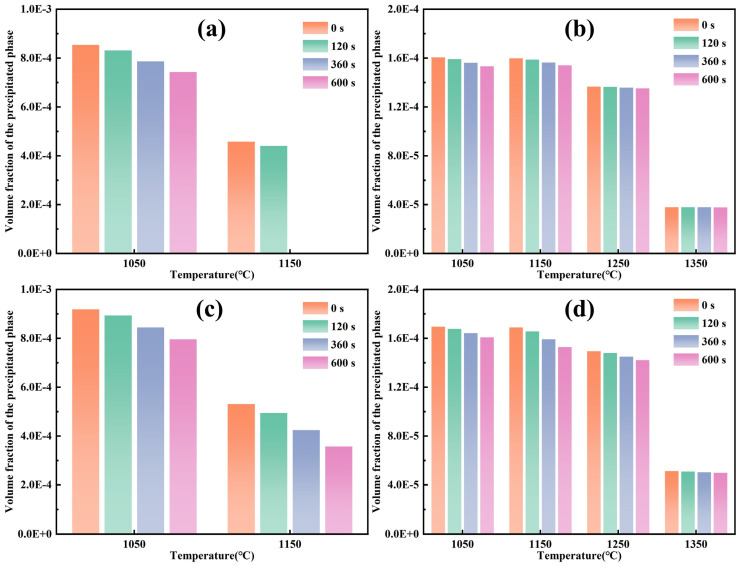
Volume fraction of precipitated phases during heating of Ce-free (**a**,**b**) and Ce-treated (**c**,**d**) steel: (**a**,**c**) Nb-rich (Nb, Ti)(C, N); (**b**,**d**) Ti-rich (Nb, Ti)(C, N).

Figure 17 presents the calculated volume fractions of Nb-rich and Ti-rich carbonitrides during the heating process in Ce-free and Ce-treated steel. During the heating process, the volume fractions and variation trends of precipitated phases in Ce-free and Ce-treated steel were essentially consistent. During holding at 1050 and 1150 °C, the dissolution of Nb-rich carbonitrides in the matrix dominated, with their volume fraction decreasing significantly as holding temperature increased and holding time extended, while the volume fraction of Ti-rich carbonitrides remained nearly unchanged. When the temperature rose to 1250 °C and above, the Nb-rich carbonitrides dissolved completely. The remaining Ti-rich carbonitrides began to dissolve substantially with increasing temperature, resulting in a marked decrease in their volume fraction. These calculated results were consistent with the TEM images shown in Figure 13 and Figure 14. However, the effect of holding time on them remained relatively minor. At 1150 °C, the dissolution behavior of precipitated phases in the two experimental steels differed. After holding at 1150 °C for 360 and 600 s, the volume fraction of Nb-rich carbonitrides in Ce-free steel was 0, indicating complete dissolution, which corresponded with the observations in Figure 13e,h. In contrast, after holding for 600 s, the volume fraction of Nb-rich carbonitrides in Ce-treated steel was 3.57 × 10^−4^, remaining present in the matrix, matching the findings in Figure 14h. Ce delayed the dissolution of Nb-rich carbonitrides at 1150 °C with the increase in holding time.

**Figure 18:**
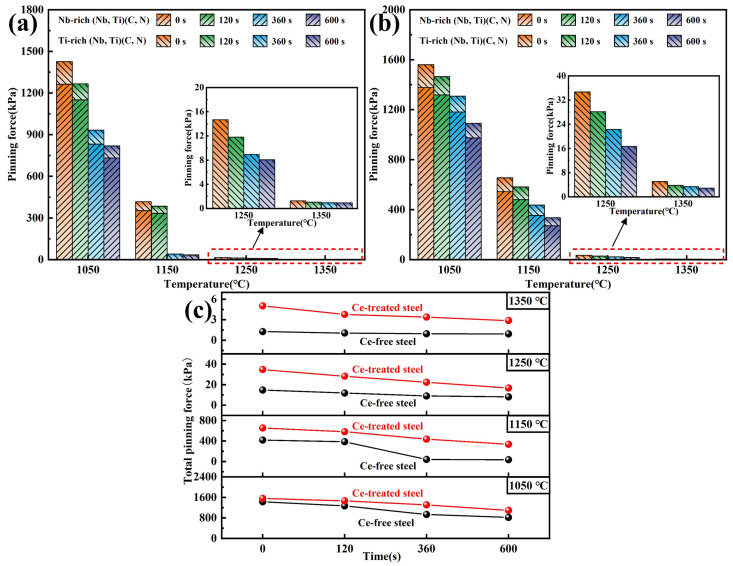
Calculation results of the pinning force of precipitated phases at grain boundaries in Ce-free (**a**) and Ce-treated (**b**) steel during heating and comparison of total pinning forces (**c**).

Furthermore, the pinning forces of precipitated phases at grain boundaries during the heating process of Ce-free and Ce-treated steel were calculated and compared, with the results shown in Figure 18. Substituting the results for the average diameter of precipitated phases from Figure 16 and the calculated volume fraction from Figure 17 into Equation (12), the pinning forces were obtained. As shown in Figure 18a,b, when held at 1050 and 1150 °C, the total pinning force was primarily provided by two precipitated phases. Due to the smaller size and higher volume fraction of the Nb-rich carbonitrides, the pinning force they provided was dominant. The decrease in total pinning force with increasing holding time was predominantly due to the dissolution of Nb-rich carbonitrides during the heating and holding process. When the holding temperature reached 1250 and 1350 °C and held for 600 s, the pinning force was entirely provided by Ti-rich carbonitrides. The remaining Ti-rich carbonitrides in the matrix rapidly dissolved and coarsened, significantly weakening their pinning effect on grain boundaries and causing a sharp decrease in the total pinning force. From Figure 18c, after adding 0.0070% Ce, the total pinning force was consistently higher than that in Ce-free steel under identical heating conditions. At 1050 °C, the total pinning force of Ce-treated steel was slightly higher than that of Ce-free steel due to its marginally smaller precipitated phase size. When held at 1150 °C for 360 s or longer, both precipitated phases in Ce-treated steel continued to pin grain boundaries, whereas only Ti-rich carbonitrides remained in Ce-free steel, widening the gap in total pinning force between the two steels. At 1250 and 1350 °C, the Ti-rich carbonitrides in Ce-treated steel were significantly smaller than those in Ce-free steel, and the total pinning force was markedly higher than that of Ce-free steel.

**Figure 19:**
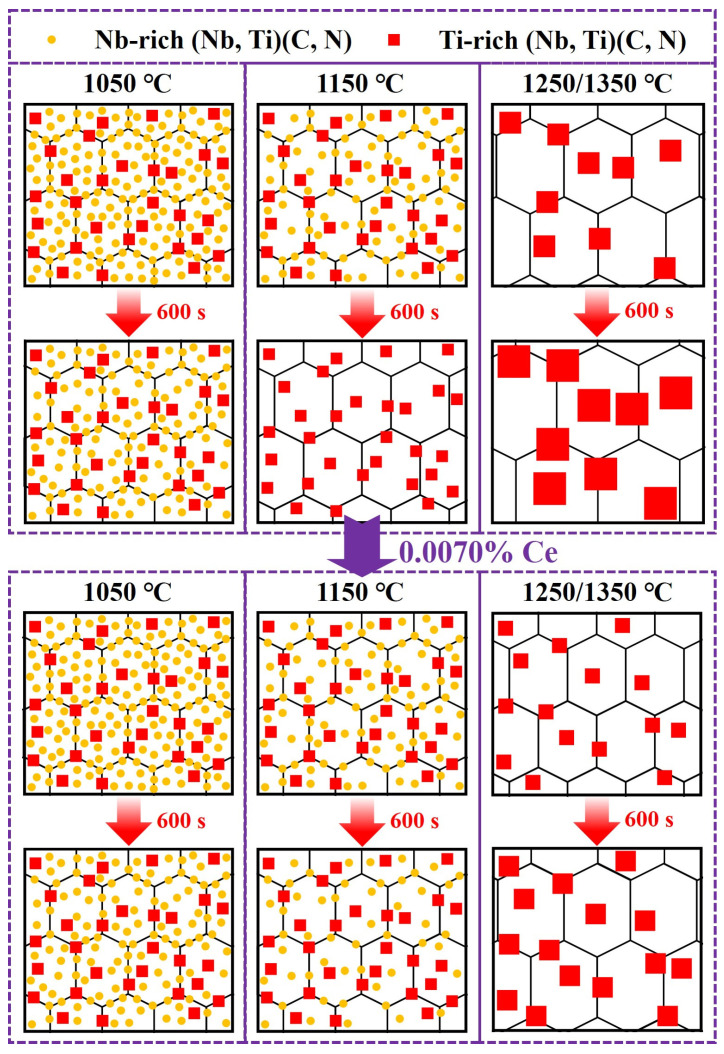
Mechanism diagram of Ce refining austenite grain during heating process.

From the above analysis, the mechanism by which Ce refined the austenite grain size during heating process was summarized, as shown in Figure 19. When held at 1050 °C, the total pinning force remained at a relatively high level due to the pinning effects provided by two types of precipitates on the grain boundaries in Ce-free and Ce-treated steel. Consequently, the grain growth rate was low for both steels, resulting in similar grain sizes, and Ce had little effect on grain growth. At 1150 °C, the complete dissolution of Nb-rich carbonitrides in Ce-free steel after 360 s of holding significantly reduced the total pinning force, substantially weakening the pinning effect on grain boundaries. This resulted in a substantial increase in grain size with extended holding time. During the holding process of Ce-treated steel, both precipitated phases remained present, maintaining a high total pinning force that effectively impeded grain boundary migration. Grain growth proceeded at a relatively slow rate, resulting in a greater size disparity compared to Ce-free steel. At holding temperatures of 1250 and 1350 °C for holding, only large-sized Ti-rich carbonitrides remained in steel. These carbonitrides rapidly coarsened and dissolved, leading to a significant reduction in total pinning force. This failure to continue suppressing grain boundary migration resulted in rapid grain growth. Since the Ti-rich carbonitrides in Ce-treated steel were significantly smaller than those in Ce-free steel, they provided higher pinning force, resulting in finer grains. Therefore, during the heating and holding process, Ce enhanced the pinning ability of precipitated phases at grain boundaries by refining the precipitates and delaying their dissolution, thereby playing a crucial role in grain refinement.

**Figure 20:**
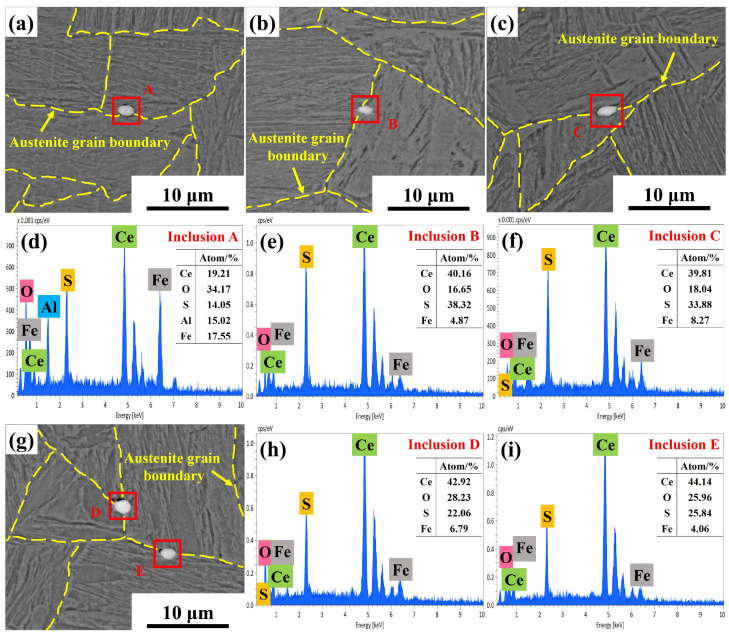
Rare earth inclusions at the grain boundaries of Ce-treated steel during the heating process: (**a**,**d**) CeAlO_3_ + Ce_2_O_2_S; (**b**,**c**,**e**–**i**) Ce_2_O_2_S.

Additionally, numerous rare earth inclusions were observed at the grain boundaries of Ce-treated steel, as shown in Figure 20. The majority of rare earth inclusions at grain boundaries were Ce_2_O_2_S, with a small amount of CeAlO_3_ + Ce_2_O_2_S inclusions also observed, all of which were small in size. The number density and average size of CeAlO_3_ + Ce_2_O_2_S complex inclusions were 0.56 per mm^2^ and 2.02 μm, respectively, whilst those of Ce_2_O_2_S inclusions were 10.16 per mm^2^ and 2.28 μm, respectively. The melting points of CeAlO_3_ and Ce_2_O_2_S are 2031 and 1950 °C, respectively, far exceeding the holding temperatures, allowing them to remain stable throughout the heating process. Compared to Ce-free steel, the addition of 0.0070% Ce resulted in the formation of numerous fine Ce_2_O_2_S and CeAlO_3_ rare earth inclusions distributed at the austenite grain boundaries during heating and holding. These inclusions acted as pinning sites for the grain boundaries, effectively inhibiting grain growth [40,41]. Thus, Ce not only enhanced pinning force by interacting with the precipitated phases, but the formation of rare earth inclusions further increased pinning force at grain boundaries, which also contributed to grain refinement during heating.

Although existing research has demonstrated that Ce could effectively inhibit grain growth during the heating process, particularly at high temperatures, experimental steel subsequently undergoes TMCP rolling and heat treatment, during which grains continue to exhibit hot deformation, dynamic recrystallization, and growth. Therefore, the current research still has certain limitations. Owing to the heredity of the microstructure, the finer grains obtained in Ce-treated steel after heating also exert a positive influence on the final microstructure and mechanical properties. The specific effects require further analysis to enhance the practical value of Ce treatment in research concerning the microstructure and properties of high-strength low-alloy steel.

## 4. Conclusions

Based on in situ observations of grain growth behavior during the heating process of high-strength low-alloy steel, combined with dynamic analysis and precipitated phase characterization, this study comprehensively investigated the effects of heating parameters and Ce on grain growth, clarifying the specific mechanism by which Ce refined the austenite grains. The main conclusions are as follows:

(1) After adding Ce, the grain coarsening temperature increased from 1050–1150 °C to 1150–1250 °C, and grain refinement occurred during heating. After 600 s of holding at 1050 °C, the average grain sizes of Ce-free and 0.0070% Ce steel increased by only 3.44 and 3.67 μm, respectively, demonstrating strong resistance to grain coarsening. At this temperature, Ce had little effect on grain growth. At temperatures of 1150 °C and above, the grain size of 0.0070% Ce steel was smaller than that of Ce-free steel. When the holding time was 600 s, the average grain sizes differed by 18.54%, 27.75%, and 21.50%, respectively.
(2) Dynamic models of austenite grain growth during the heating and holding process were established for Ce-free steel and 0.0070% Ce steel, denoted as ${D_{t}}^{2}-{D_{0}}^{2}=4902907.8755t\exp\left(-\frac{172058.23}{RT}\right)$ and ${D_{t}}^{2}-{D_{0}}^{2}=19218730.7371t\exp\left(-\frac{193702.8976}{RT}\right)$, respectively. After treatment with 0.0070% Ce, the activation energy for grain growth increased by 21.65 kJ/mol, making grain growth more difficult.
(3) During the holding temperature range, the precipitated phases were all (Nb, Ti)(C, N). Without Ce addition, when held at 1050 °C, a large amount of small-sized Nb-rich carbonitrides were present, exhibiting high total pinning force and slow grain growth. At 1150 °C, the Nb-rich carbonitrides completely dissolved with increasing holding time, leading to a substantial decrease in total pinning force and a marked increase in grain size. Upon heating to 1250 °C and above, the remaining Ti-rich carbonitrides coarsened and dissolved extensively, resulting in rapid grain coarsening.
(4) During heating, compared to Ce-free steel, the 0.0070% Ce content refined the size of precipitated phases, delayed the dissolution of Nb-rich carbonitrides during holding at 1150 °C, and generated numerous small-sized high-melting-point Ce_2_O_2_S and CeAlO_3_ inclusions. This enhanced pinning forces at grain boundaries, making grain boundary migration more difficult and effectively inhibiting grain growth.

## Figures and Tables

**Table 1:** Chemical compositions of experimental steels (wt%).

Sample	C	Si	Mn	V	Ti	Nb	Al	O	S	B	Ce	Fe
Ce-free	0.12	0.29	1.57	0.069	0.015	0.046	0.015	0.0022	0.0046	0.0017	0	Bal.
Ce-treated	0.12	0.29	1.62	0.073	0.017	0.047	0.018	0.0012	0.0029	0.0019	0.0070	Bal.

## Data Availability

The original contributions presented in this study are included in the article. Further inquiries can be directed to the corresponding author.
